# Post‐COVID immunity in patients with solid tumor or hematological malignancies treated with SARS‐CoV‐2 monoclonal antibodies

**DOI:** 10.1002/iid3.70039

**Published:** 2024-12-10

**Authors:** Gilberto Sabino‐Santos, Cathryn E. Leggio, Sean M. Litwin, Najia Waheed, Shuangyi Bai, Sinem Ulusan, Anoli Karunathilake, Debra H. Elliott, Ashley R. Smira, Sruti Chandra, Lin Li, Bo Ning, Tony Hu, John S. Schieffelin, Bronwyn M. Gunn, James E. Robinson, Jyotsna Fuloria, Elizabeth B. Norton

**Affiliations:** ^1^ Department of Microbiology and Immunology Tulane University School of Medicine New Orleans Louisiana USA; ^2^ University Medical Center New Orleans New Orleans Louisiana USA; ^3^ Paul G. Allen School of Global Health Washington State University Pullman Washington USA; ^4^ Department of Pediatrics Tulane University School of Medicine New Orleans Louisiana USA; ^5^ Department of Biochemistry and Molecular Biology Tulane University School of Medicine New Orleans Louisiana USA

**Keywords:** hematological malignancies, memory immunity, monoclonal antibody therapy, SARS‐CoV‐2

## Abstract

**Purpose:**

SARS‐CoV‐2 monoclonal antibody (mAB) therapy has effectively treated severe COVID‐19, although how this contributes to protective antiviral immunity in settings of malignancy is poorly defined.

**Patients and Methods:**

We evaluated the development of post‐infection immunity in five patients with malignancies who received mAB therapy targeting spike protein for their PCR‐confirmed SARS‐CoV‐2 infection in 2021, compared with non‐mAB controls. Patients were identified from a larger study on oncology with a history or documented current infection with SARS‐CoV‐2. Subjects include two patients with lymphoma and CD20‐depletion therapy, one with myeloma and two with solid tumor (stage IIA rectal adenocarcinoma and metastatic breast cancer). Cancer therapies and COVID vaccination history varied by patient. Blood samples (1–4 per patient) were collected 71–635 days post‐mAB therapy. We employed clinical histories with comprehensive immunoprofiling analysis, including systems serology antibody isotyping and effector function, T‐cell immunophenotyping for subset and memory cells, and sensitive blood viral RNA detection up to 2 years post‐mAB therapy.

**Results:**

B‐cell deficiency was confirmed in 3/5 patients. All patients had detectable anti‐spike and nucleoprotein antibody isotypes, effector functions, and neutralizing antibodies (which increased over time by subject) at similar levels to the control group. Virus‐specific T‐cell activation and phenotypes varied by time and patient. Spike‐specific effector and memory CD8 + T‐cells were significantly elevated in mAB subjects compared to the control group. SARS‐CoV‐2 viral RNA detection was also higher in mAB‐treated patients. One patient on bortezomib therapy had unique alterations in these populations.

**Conclusion:**

All mAB‐treated patients with malignancies developed polyfunctional immunity humoral and T‐cell immunity to SARS‐CoV‐2 even in the setting of B‐cell deficiency. The evolution of this immunity, including new variant‐specific antibodies, without secondary illnesses suggests that patients were protected from symptomatic re‐infection, and mAB therapy did not blunt the development of host immunity. Future studies are warranted to better characterize immunologic memory over time with exposures to new viral variants, evaluate prolonged viral shedding and the continued use of appropriate mAB for infection in high‐risk patients.

## Graphical abstract

Utilizing in‐depth immunoprofiling of patients with cancer who received mAB therapy against SARS‐CoV‐2 infection, we showed that all patients had polyfunctional viral immunity irrespective of mAB therapy or B‐cell depletion with evidence of asymptomatic reinfection.

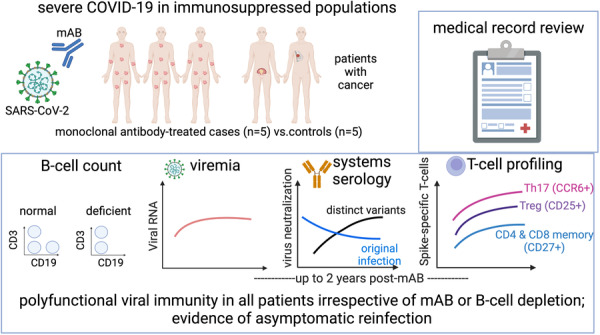

## 1 INTRODUCTION

Severe acute respiratory syndrome coronavirus 2 (SARS‐CoV‐2) is the cause of the pandemic coronavirus disease 2019 (COVID‐19).^1^ SARS‐CoV‐2 is an enveloped, single‐stranded, positive‐sense RNA virus. Spike (S)‐protein and other essential viral structural proteins, such as the nucleocapsid (N), membrane (M), and envelope (E), are the primary determinants of virulence and function.^2^ Infections can reach the lower respiratory tract^3^ and progress to pneumonia. Acute respiratory distress syndrome, organ failure and death can occur due to extreme immune responses.^4^ Patients with cancer are considered at high risk for severe infection outcomes due to weakened immune systems from chemotherapy or other immunosuppressive regimens. Patients with hematologic malignancies have a higher risk of prolonged infection and death from COVID‐19 than patients with solid tumors, typically due to lower levels of antibodies, T‐cell exhaustion, and use of B‐cell‐depleting therapies.^5^

Vaccines were developed for SARS‐CoV‐2, including mRNA vaccines targeting the S protein generating antibodies that block viral entry.^2^ Additional therapies for COVID‐19 have included antiviral therapies, anti‐inflammatory agents, and targeted passive immunotherapy with neutralizing recombinant monoclonal antibodies (mAB).^1^ In November 2020, emergency use authorization (EUA) for mABs included bamlanivimab (B‐mAB) (from November 2020‐April 2021) and casivirimab‐imdevimab (C/I‐mAB) (November 2020‐January 2022). However, EUA was revoked for these due to reduced efficacy against the Omicron variant. mAB therapy against SARS‐CoV‐2 was associated with improved clinical outcomes for patients with cancer early in the pandemic.^6^, ^7^ Most studies have reported only on short‐term mAB clinical outcomes. One recent study characterized the development of T‐cell responses with B‐mAB in 46 subjects, although not specific to patients with cancer.^6^ Immunocompromised individuals, such as patients with hematological malignancies, are at risk for more severe COVID‐19 outcomes,^8^ with need for additional preventive or therapeutic measures beyond the current vaccination programs.

Thus, there has been a limited understanding of post‐infection mAB immunity in specific populations with highly diverse medical histories over time. Therefore, here we performed in‐depth immunoprofiling of antiviral responses in patients with hematological or solid tumor malignancies who were treated with SARS‐CoV‐2 mAB for COVID‐19 from New Orleans, LA, in 2021, followed up to 2 years.

## 2 METHODS

### 2.1 Study population

Following the Good Clinical Practice guidelines, patients were enrolled under the protocol (#1838) FWA# A00002762. Patients were identified at the University Medical Center New Orleans Cancer Center or inpatient oncology floor by specific prescreening criteria as part of a larger study consisting of cancer diagnosis in combination with history or a documented current infection with SARS‐CoV‐2. The inclusion criteria consisted of a cancer diagnosis in combination with a current or previous PCR‐confirmed SARS‐CoV‐2 infection. Blood samples were selected from this larger study for patients receiving mAB therapy (*n* = 5). We also included samples of five patients with solid tumor and lymphoma diagnosis and previous SARS‐CoV‐2 infection as reference controls who had not received mAB therapy for COVID‐19.

### 2.2 Blood processing and viral detection

Blood was processed for PBMCs and plasma isolation. Viral RNA load was measured by SARS‐CoV‐2‐specific CRISPR assay targeting the nucleocapsid gene in heat inactivated plasma.^9^

### 2.3 Antibody and t‐cell analyses

Plasma samples were evaluated for viral antibodies, including by ELISA, neutralization, multiplex assay for antibody isotypes and Fc‐mediated effector functions.^10^ PBMCs were tested by Activation‐Induced Marker (AIM) on 24h‐restimulated cells^11^ with viral peptide pools (Figure S1, Table S2).

### 2.4 Data

Data were generated using GraphPad v. 9.5.1, JMP Pro v.16.2.0, R Studio v.4.1.2., Adobe Illustrator v. 27.2, FlowJo v10.8.2 (BD Company), BioRender, Cytoscape v.3.10.0, or obtained from https://ldh.la.gov/coronavirus/. All data are available upon request to EBN. Detailed methods are provided in Supplemental Material and all patient and sample data is included in Supplemental Data spreadsheet.

## 3 RESULTS

### 3.1 Patient characteristics

**Figure 1:**
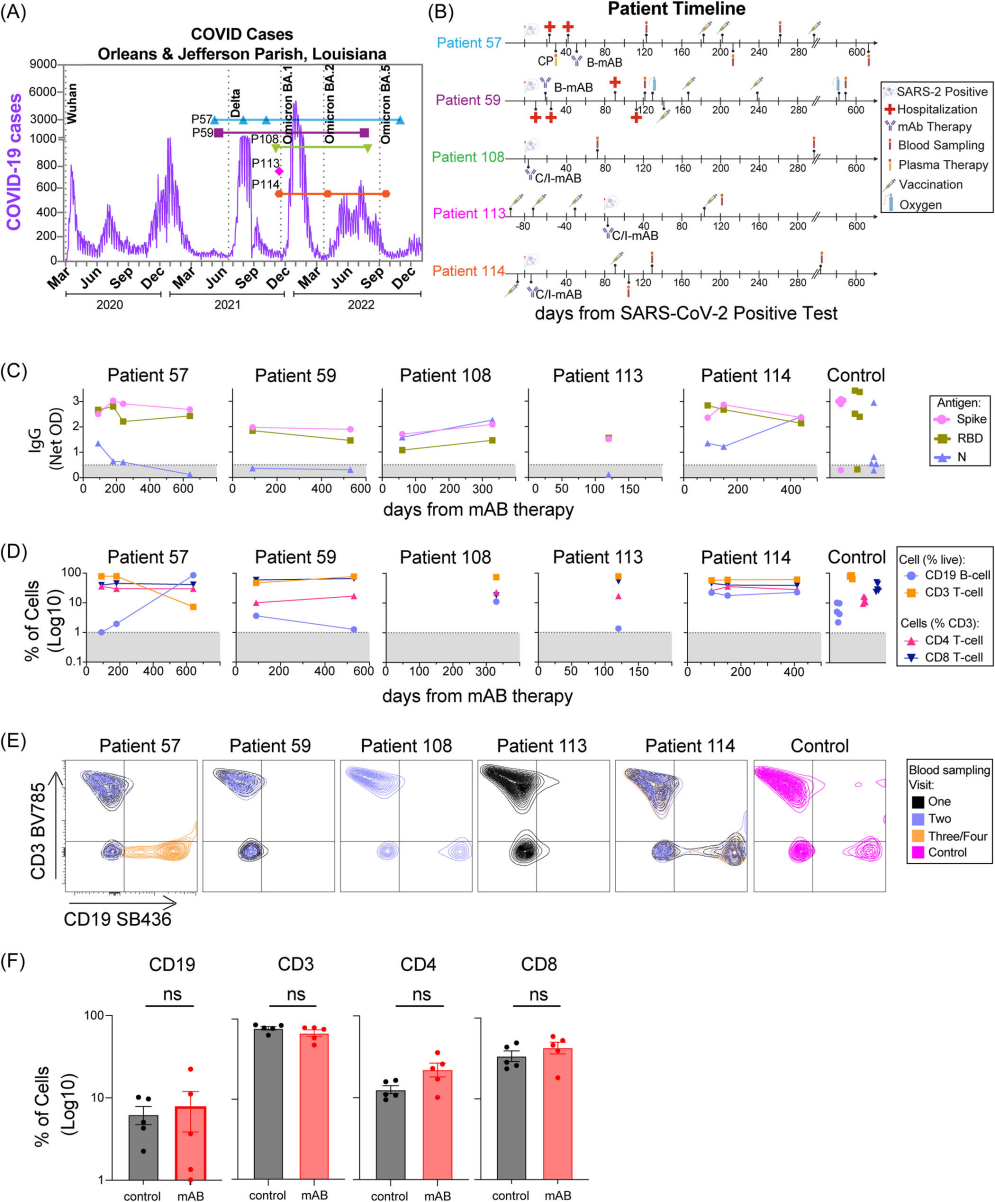
Timeline of each patient sample collection and patient‐specific changes in S, N, or RBD IgG or % B‐cells. (A) Timeline of each patient sample according to COVID case levels in Orleans and Jefferson Parishes in Louisiana with Wuhan, Delta, and Omicron variants peaks indicated. (B) Patient‐specific timelines for infection, treatments, and blood sample visits by days from SARS‐CoV‐2 positive PCR tests. Treatments included bamlanivimab (B‐mAB), casirivimab‐imdevimab (C/I‐mAB), and convalescent plasma (CP), as indicated. (C) ELISA IgG antibodies against S, RBD, or N SARS‐CoV‐2 antigens as indicated. (D) Flow cytometry percentage of CD19 B‐cells or CD3 T‐cells recovered from live‐gated PBMCs (postfreeze/thaw) or CD4 T‐cells and CD8 T‐cells from CD3 T‐cells. The Gray patterned area indicated the limit of detection for each assay on C and D. (E) Contour plot of CD3 T‐cells vs CD19 B‐cells from each patient per blood sampling visit 1–4. (F) Comparison of CD3 T‐cells, CD19 B‐cells from mAB‐treated patients vs control group. All comparisons were performed between the first‐time point sample of each sample for mAB‐treated patients versus the control group with unpaired non‐parametric *t*‐tests.

The study involved patients with diverse cancer types undergoing various immunosuppressive therapies with samples collected after Wuhan and Delta viral strain infections during 2021–2022 (Figure 1A, additional details in the supplemental material). Five patients (57, 59, 108, 113, and 114) were treated with B‐mAB or C/I‐mAB for severe SARS‐CoV‐2 infection, and samples were collected 71–615 days later (Figure 1B **).** Vaccination histories varied, with all subjects receiving monovalent mRNA vaccines (except for subject 108, unvaccinated) (Figure 1B and supplemental material). Two patients (57 and 59) with B‐cell lymphoma received B‐mAB against SARS‐CoV‐2, showing distinct antibody responses and B‐cell dynamics (Figure 1C–E). Three patients with distinct cancer diagnoses (108 with stage II A rectal adenocarcinoma, 113 with metastatic breast cancer, and 114 with multiple myeloma) received C/I‐mAB, exhibiting patient‐specific antibody profiles and B‐cell levels (Figure 1C–E). Despite these differences, 4 out of 5 patients experienced a favorable clinical course within 120 days post‐infection, without requiring subsequent hospitalization up to 665 days post‐infection. One patient (59) had Acute respiratory distress syndrome (ARDS) and was re‐hospitalized 119–133 days post‐mAB and was then discharged with prolonged corticosteroid treatment and supplemental oxygen (Figure 1B). These 5 patients were also compared to a control cohort of previously infected cancer patients (one lymphoma, 4 solid tumors) not receiving mAB. No differences were observed in % of lymphocyte populations in recovered PBMCs between mAB‐treated patients and controls (Figure 1F).

### 3.2 Evolution of antibody responses and re‐infections in mAB‐treated patients

**Figure 2:**
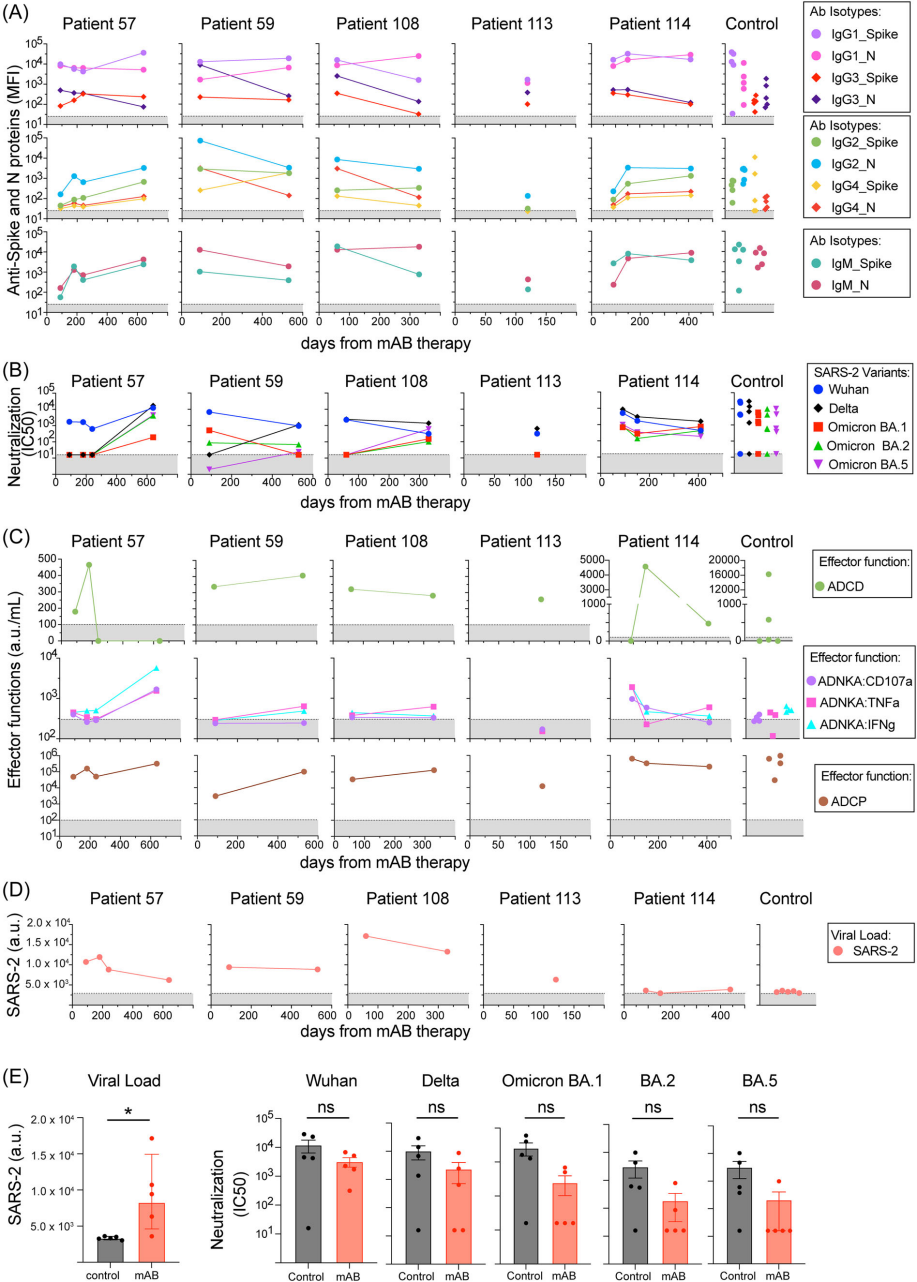
mAB‐treated patients display evidence of evolving antibody magnitudes and functions and viral load post‐mAB therapy for SARS‐CoV‐2 infection. (A) Multiplex detection of IgG1‐4 or IgM isotypes against S or N SARS‐CoV‐2 antigens was reported as mean fluorescent intensity (MFI). (B) Neutralizing antibodies against SARS‐CoV‐2 Wuhan or Delta and Omicron BA.1, BA.2, BA.5 variants. (C) Spike‐specific fc effector functions, including ADCD, ADCD, ADNKA (CD107a, TNF‐α, or IFNγ induced change), or ADCP, all reported as arbitrary units per mL of plasma (a.u./mL). (D) Viral load detection of SARS‐CoV‐2 N RNA by sensitive CRISPR assay on plasma samples, reported as arbitrary units (a.u.) of fluorescent intensity. (E) Comparison of viral load and neutralizing antibodies from mAB‐treated patients vs control group. The Gray patterned area indicated the limit of detection for each assay. All comparisons were performed between the first‐time point sample of each sample for mAB‐treated patients versus the control group with unpaired non‐parametric *t*‐tests.

All patients had detectable anti‐S and ‐N antibodies 71–635 days post‐mAB treatment (Figure 1C), at levels similar to the control group (Figure S1A). We profiled patient samples for antibody subclasses (IgG1, IgG2, IgG3, IgG4, and IgM), noting that vaccination and infection can alter anti‐S responses, while infection alone elicits anti‐N responses (Figure 2A, S1B‐C). We observed evidence of high levels of anti‐S IgG1 for all patients, the same isotype and antigen target of the B‐mAB and C/I‐mAB, similar to control subjects. These IgG levels remained detectable for all mAB‐treated patients over time. Viral‐specific IgG3 was also detected following similar patterns to IgG1. In contrast, anti‐S or anti‐N IgG2 or IgG4 increased from the first blood collection to the subsequent blood samples in mAB‐treated patients (excluding patient 113, who had a single blood sample). Anti‐S and anti‐N IgM, often a measure of active or recent infection, were also detected, with anti‐N IgM increasing over time in mAB‐treated patients 57, 108, 114 (Figure 2A, S1D). No differences were observed between antibody isotypes of mAB‐treated and control patients (Figure S1).

Following, we evaluated neutralization and Fc‐mediated innate immune effector functions (antibody‐dependent complement deposition [ADCD], antibody‐dependent natural killer cell activation [ADNKA] and antibody‐dependent cellular phagocytosis [ADCP]) (Figure 2B‐C, Figure S1B). In these assays, purified B‐mAB had high levels of Wuhan‐specific neutralization and spike ADCD and ADCP but not ADNKA (Table S1). Strain‐specific neutralization (Delta or Omicron BA.1, BA.2, BA.5) increased in 3/4 mAB subjects with multiple collection time points, while Wuhan‐specific neutralizing antibodies declined. Similar responses were observed between mAB and control subjects, except for Omicron neutralization (*p* = *0.0635*) (Figure 2E). Changes in neutralizing antibody profiles to variants are linked to secondary infections with SARS‐CoV‐2 variants.^12^ ADNKA (using CD107a, TNF‐α, and INFγ expression) and ADCP also changed in mAB subjects over time (Figure 2C, E), but with no significant differences to the control group (Figure S1B). ADCD followed mAB patient‐specific patterns over time. Lastly, SARS‐CoV‐2 RNA (not variant specific) was detected in all mAB‐treated patients with significant differences when compared to the control group (Figure 2E). These data indicate that all mAB‐treated patients in this study had an evolution of their SARS‐CoV‐2 antibodies and effector functions 1–2 years post‐initial infection with minimal or no significant differences compared to the control group, except for viral RNA, concurrent with evidence of new SARS‐CoV‐2 variant exposure.

### 3.3 Viral‐specific t‐cell immunity after mAB‐treated infections

**Figure 3:**
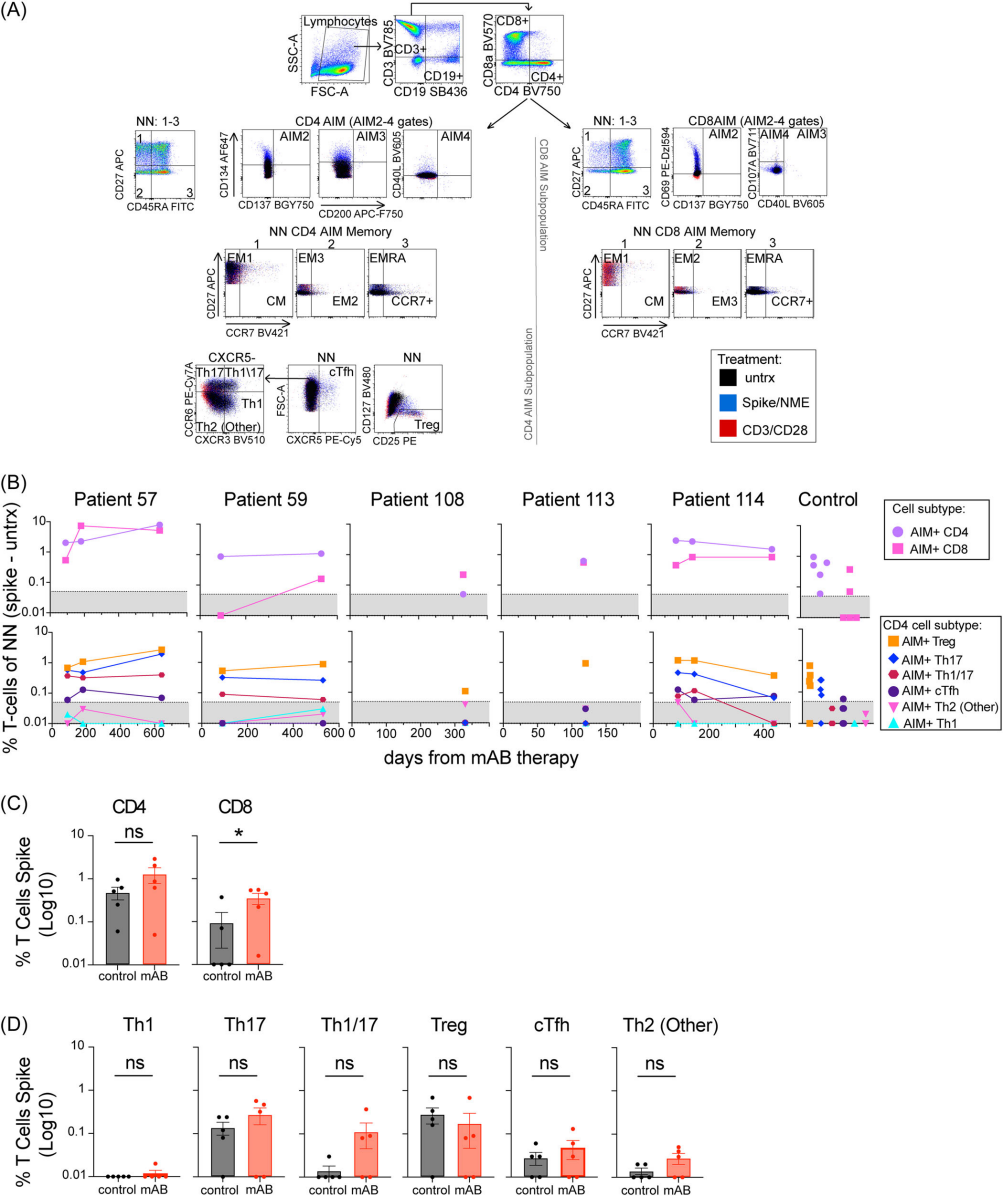
mAB‐treated patients developed spike‐specific AIM + CD4 and CD8 T‐cells, Treg, and Th17 subsets. (A) Dot blots and gating strategy for AIM panel identifying CD4 and CD8 cells in Non‐Naïve (NN, all T‐cells not CD27 + CD45RA+) expressing surface activation markers (AIM 2‐4 gates with AIM+ identified as any 2/4 activation markers expressed), CD4 T‐cell subsets (Th1, Th17, Th1/17‐like, Th2, Treg, cTfh) or CD4 or CD8 memory markers (EM1‐3, CM, EMRA). Unstimulated (untrx), spike, or non‐spike (NME) peptide pools were used for 24 h stimulation of PBMCs in this panel. (B) Normalized AIM + CD4 or CD8 AIM T‐cell, or AIM + CD4 subsets Th1, Th1/Th17, Th17, Th2 (Other), Treg, cTfh as % NN. The Gray patterned area indicated the limit of detection for AIM assay. (C) Comparison of AIM + CD4 and CD8 T‐cells from mAB‐treated patients vs control group. (D) Comparison of AIM + CD4 subsets from mAB‐treated patients vs control group. All comparisons were performed between the first‐time point sample of each sample for mAB‐treated patients versus the control group with unpaired non‐parametric *t*‐tests.

We next tested patients for virus‐specific T‐cell responses using AIM assay, which measures the expression of any two activation markers (CD143, CD137, CD200, CD40L on CD4 T‐cells or CD69, CD137, CD107A, CD40L on CD8 T‐cell). Viral‐specific AIM + CD4 and CD8 T‐cells have been detected after vaccination and infection.^6^, ^11^, ^13^ These cells were immunophenotyped for CD4 T‐helper‐1 (Th1), Th2, Th17, T‐follicular helper (Tfh), circulating (c)Tfh, and T‐regulatory (Treg) subsets (Figure 3A and Figure S2) based on the expression of CXCR5, CXCR3, CD25, and CCR6. We observed evidence of AIM + CD4 and CD8 T‐cells specific to spike or non‐spike (NME) peptide pools from all mAB‐treated patients and controls (Figure 3B, S2B, S3A). Notably, viral‐specific AIM + T‐cells were detected in samples from visits one and two of patients 57 and 59 (Figure 3B, S3A, S4) with lymphoma and B‐cell deficiency (Figure 1B‐D). AIM + CD8 T‐cells were also detected in unvaccinated patient 108 (Figure 3B). mAB‐treated patients had significantly higher spike‐specific AIM + CD8 spike than control (Figure 2C).

Spike‐specific AIM + CD4 and CD8 T‐cells levels in patients with multiple samples changed similarly to antiviral antibodies (e.g., increasing in patients 57, 59, and decreasing in patient 114) (Figures 1, 2). In all samples, AIM + CD4 T‐cells were predominantly CD25+ Treg and CXCR3 + Th17 subsets, and similar between the mAB and control group (Figure 3D, S4). Past reports on severe COVID‐19 also detected AIM + CD4 Treg and Th17 subsets, including one study on B‐mAB post‐therapy T‐cell immunity in 46 subjects (unspecified for malignancies).^6^, ^14^ Similar NME‐specific AIM + T‐cells and Spike‐specific intracellular cytokines subset profiles were observed in patients 57 and 114 (Figure S3). T‐cell immunity developed post‐infection in all mAB‐treated patients, even those with significant B‐cell deficiency.

### 3.4 Evidence for t‐cell memory in mAB‐treated patients

**Figure 4:**
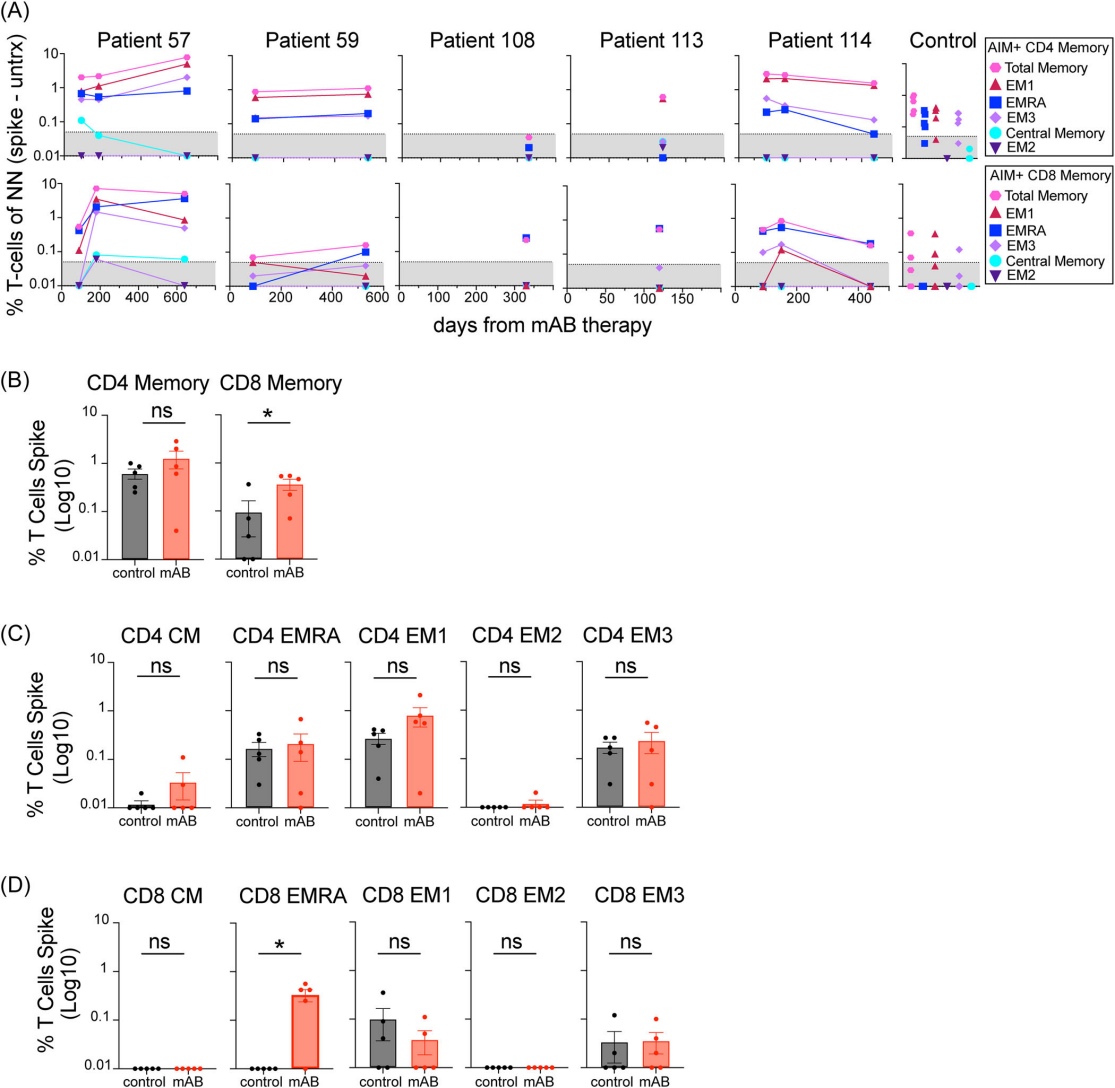
mAB‐treated patients develop spike‐specific AIM + CD4 and CD8 T‐cells memory populations correlating with vaccination. (A) Normalized AIM + CD4 or CD8 T‐cell memory populations, including total memory, EMRA, EM1, EM2, EM3, and CM subsets. The Gray patterned area indicates the limit of detection for AIM assay. (B) Comparison of AIM + CD4 and CD8 T‐cell memory (CD27+ , CD27‐ CD45RA‐, CD45RA+) from mAB‐treated patients versus control group. (C and D) Comparison of AIM + CD4 and CD8 T‐cells memory subsets from mAB‐treated patients vs control group. All comparisons were performed between the first‐time point sample of each sample for mAB‐treated patients versus the control group with unpaired non‐parametric t‐tests.

Differentiation of CD4 and CD8 T‐cell effector and memory cells is observed with antigen exposure, including after SARS‐CoV‐2 infection and vaccination.^11^, ^15^, ^16^ These responses can be defined as effector memory 1 (EM1), EM2, EM3, terminally differentiated effector memory (EMRA), and central memory (CM) based on the expression of CD27, CCR7, and CD45RA (Figure 3A). Memory states on spike‐specific AIM + T‐cells from patients and controls were observed, with a significant difference between total AIM + CD8 memory T‐cells (Figure 4A‐D). Central memory T‐cells were not prominent, likely based on the evaluation of blood versus tissue samples. EM1 CD4 T‐cells and EMRA CD8 T‐cells were the principal memory phenotypes observed in all patients, with significant differences between mAB and control group in EMRA CD8 T‐cells (Figure 4A‐D). We conclude that viral‐specific AIM + CD8 T‐cells were distinguishable in mAB‐treated patients by their memory state in comparison with controls.

## 4 DISCUSSION

We report in‐depth immunological profiles of five patients with hematological or solid malignancies who received spike‐targeting mAB therapy for severe COVID‐19. The patients received mAB therapy, which was effective during Wuhan (B‐mAB) and Delta (C/I‐mAB) infection peaks. All patients exhibited evidence of viral‐specific antibodies and T‐cell memory up to 2 years post‐mAB therapy. No patient reported a second severe or symptomatic re‐infection during the sampling time, similar to a recent study where patients who received B‐mAB had a lower probability of hospitalization compared with patients who did not receive mAB therapy.^17^

Patients' virus‐specific antibodies and T‐cells evolved or were maintained in sequential collections. This agrees with a recent meta‐analysis.^18^ We detected significant viral RNA in patient samples and higher spike‐specific effector and memory CD8 T‐cells in mAB patients. This could be a sign of multiple infections or evidence of sustained infection, as reported elsewhere.^12^ Regardless, the changes in viral‐specific antibody isotypes (N‐specific IgM) and effector functions (variant‐specific neutralization, although not unique between mAB and control), indicate that re‐infection with Omicron or other variants occurred in at least 4/5 of these patients (particularly as none of them received a bivalent booster vaccine). One study reported that mAB therapy before COVID mRNA vaccination diversifies memory B‐cells and viral‐specific immunity.^19^ Our results support this idea, as we found evidence for the continued evolution of humoral immunity to non‐spike antigens and other variants, particularly in patients 57 and 59, who both received vaccination after mAB therapy. B‐cell depletion is generally associated with recurrent SARS‐CoV‐2 infections and poor viral clearance.^20^ In B‐cell‐depleted patients, studies have shown that robust or potent CD4 T‐cell responses are more important in viral clearance than CD8 T‐cell responses,^20^ therefore protecting these patients from reinfection. We also observed high levels of AIM + CD4 T‐cell responses in mAB, but interestingly, only AIM + CD8 T‐cells were significant compared to controls. However, CD4 subsets effector and memory differentiation states of these AIM + T‐cells were comparable to other reports,^6^, ^14^, ^16^ including the detection of AIM + CD4 Treg, Th17, EM1 and CD8 T‐cell EMRA cells.

Virus‐specific immunity was detected in all patients, although we observed patient‐specific changes in serial samples. These likely are related to individual types of malignancy and infection/vaccination history. Studies on the role of mAB and SARS‐CoV‐2 immunity remain challenging due to the rapid evolution of SARS‐CoV‐2 variants and the efficacy of mAB passive immunotherapy. B‐mAB or C/I‐mAB used in this study respectively during the Wuhan and Delta variant infections ended after Omicron variant became widespread (and subsequent revoking of their EUA by the FDA). Resistance to mAB neutralization is observed even in variants with single amino acid substitutions in the spike protein^21^ and has been challenging to the implementation and use of COVID mAB therapies.

Our study has several limitations, including the small sample size and the heterogeneity of the patients in terms of malignancy types, immunosuppressive therapies, and vaccination history. Therefore, this study cannot be used to conclude any differences in SARS‐CoV‐2 immunity between patients with malignancy, by anti‐CD20 or bortezomib treatment status, or between phases of COVID‐19 (acute, etc.). While we evaluated neutralizing antibodies to variants, we did not confirm infectious viral variants or perform T‐cell epitope analyses (as due to cross‐reactive epitopes, our peptide pools would be reactive to viral variants circulating at the time of this study^22^). Therefore, the different treatment regimens and timelines of when mAB therapy was conducted make drawing direct comparisons between patients challenging. Even with the limitations of our study, we observed interesting findings that could be more carefully investigated in future studies. This includes that patient 114 with myeloma had no B‐cell deficiency or reported vaccinations between visits; however, they exhibited higher N‐specific antibodies, Omicron neutralization, and non‐spike AIM + CD4 T‐cells at their last visit. This patient's care involved a proteasome inhibitor bortezomib, which, while impeding cancer growth, increases apoptosis sensitivity and modifies T‐cells and B‐cells.^23^ The role of bortezomib therapy on post‐infection immunity in a larger group of samples may reveal clear alterations in T‐cell and B‐cell memory.

This is the first in‐depth immunoprofiling of a case series with post‐infection immunity for patients with hematological or solid tumor malignancies receiving SARS‐CoV‐2 mAB therapy. Our data reinforces that mAB therapy did not impede the development of viral immunity and protection from severe disease following re‐infection in these five patients. Future studies with more patients and controls are warranted to better characterize immunologic memory over time with exposures to new viral variants, evaluate prolonged viral shedding and the continued use of appropriate mAB for infection in high‐risk patients.

## AUTHOR CONTRIBUTIONS

**Gilberto Sabino‐Santos:** Conceptualization; Data curation; Formal analysis; Investigation; Methodology; Software; Validation; Visualization; Writing—original draft; Writing—review and editing. **Cathryn E Leggio:** Conceptualization; Investigation; Writing—original draft; Writing—review and editing. **Sean M Litwin:** Data curation; Formal analysis; Investigation; Visualization; Writing—review and editing. **Najia Waheed:** Investigation. **Shuangyi Bai:** Investigation. **Sinem Ulusan:** Investigation. **Anoli Karunathilake:** Investigation. **Debra H Elliott:** Investigation. **Ashley R Smira:** Investigation. **Sruti Chandra:** Investigation. **Lin Li:** Investigation. **Bo Ning:** Funding acquisition; Investigation; Resources. **Tony Hu:** Funding acquisition; Investigation; Resources. **John S Schieffelin:** Funding acquisition; Investigation; Resources; Writing—review and editing. **Bronwyn M Gunn:** Data curation; Formal analysis; Investigation; Validation; Writing—review and editing. **James E Robinson:** Conceptualization; Data curation; Formal analysis; Funding acquisition; Investigation; Methodology; Project administration; Resources; Validation; Writing—review and editing. **Jyotsna Fuloria:** Conceptualization; Data curation; Formal analysis; Funding acquisition; Investigation; Methodology; Project administration; Resources; Supervision; Writing—original draft; Writing—review and editing. **Elizabeth B Norton:** Conceptualization; Data curation; Formal analysis; Funding acquisition; Investigation; Methodology; Project administration; Resources; Software; Supervision; Validation; Visualization; Writing—original draft; Writing—review and editing.

## CONFLICT OF INTEREST STATEMENT

The authors declare no conflicts of interest.

## Supporting information

Supporting information.

Supporting information.

## Data Availability

All data are available upon request to EBN.
