# Supplementary material for: Post‐COVID immunity in patients with solid tumor or hematological malignancies treated with SARS‐CoV‐2 monoclonal antibodies
Source: Immun Inflamm Dis. 2024 Dec 10;12(12):e70039. doi: 10.1002/iid3.70039 (PMC11632117; doi:10.1002/iid3.70039)
Supplement: Supplementary file 1 — Supporting information. [file IID3-12-e70039-s002.docx]

# Post-COVID immunity in patients with solid tumor or hematological malignancies treated with SARS-CoV-2 monoclonal antibodies.

# Gilberto Sabino-Santos PhD^1^, Cathryn E. Leggio MS^2^, Sean M. Litwin MS^1^, Najia Waheed MD^2^, Shuangyi Bai PhD^3^, Sinem Ulusan MS^3^, Anoli Karunathilake BS^3^, Debra H. Elliott BS^4^, Ashley R. Smira BS^4^, Sruti Chandra PhD^1^, Lin Li PhD^5^, Bo Ning PhD^5^, Tony Hu MD^5^, John S. Schieffelin MD^4^, Bronwyn M. Gunn PhD^3^, James E. Robinson MD^4^, Jyotsna Fuloria, MD^2*^, Elizabeth B. Norton, PhD^1*^

1. Department of Microbiology and Immunology, Tulane University School of Medicine, New Orleans, LA.

2. University Medical Center New Orleans, New Orleans, LA.

3. Paul G. Allen School of Global Health, Washington State University, Pullman, WA.

4. Department of Pediatrics, Tulane University School of Medicine, New Orleans, LA.

5. Department of Biochemistry and Molecular Biology, Tulane University School of Medicine, New Orleans, LA.

*Corresponding authors: Elizabeth B. Norton: 1430 Tulane Ave., SL-38, New Orleans, LA 70112-2699 tel 504.998.2205 e-mail: [enorton@tulane.edu](mailto:enorton@tulane.edu). Jyotsna Fuloria: 2000 Canal St., New Orleans, LA 70112 tel 501.702.4410 e-mail: [Jyotsna.Fuloria@lcmchealth.org](mailto:Jyotsna.Fuloria@lcmchealth.org)

**Detailed Patients Characteristics**

**Patient characteristics 57 with lymphoma receiving B-mAB***.*

A 49-year-old male with a history of resected renal cell carcinoma (2018) and small lymphocytic lymphoma (SLL) (April 2019) was treated with rituximab, a B-cell targeting anti-CD20 therapy, for four weeks in November 2019. He received ibrutinib, a B-cell proliferation inhibitor, along with rituximab but experienced disease progression accompanied by significant lymphadenopathy and fatigue in October 2020. He was diagnosed with COVID-19 post-rituximab infusion on January 15, 2021, leading to hospitalization due to respiratory failure with a diagnosis of pneumonia 20 days post-infection (d.p.i.). Treatment included convalescent plasma 30 d.p.i. (**Figure 1B**), along with ten days of dexamethasone and five days of remdesivir. Despite initial improvement, he had persistent symptoms and required re-admission (35 d.p.i.). Further treatment with remdesivir and one dose of the anti-spike B-mAB by infusion 50 d.p.i. (**Figure 1B**) resulted in symptom improvement. Cognitive deficits continued for many months post-discharge. Blood samples were collected (125, 217, 262, and 665 d.p.i.) for immunologic assays (**Figure 1B**). The patient presented high levels of anti-Spike and RBD antibodies (**Figure 1C**), likely due to B-mAB and vaccination with mRNA BNT162b2 (182, 203, and 357 d.p.i.) (**Figure 1B**). In addition, as anticipated from B-cell depletion therapy, the subject exhibited a lower % CD19 B-cells for visits 1-2, with a notable increase at visit 4 (**Figure 1D-E**).

**Patient 59 with lymphoma receiving B-mAB***.*

A 63-year-old female was diagnosed with an atypical B-cell SLL at stage IV in 2011, progressing to ocular adnexal, breast, bone, and pulmonary involvement. A lymph node pathology reported CD10-/BCL6-/MUM1+/Ki-67 50-60%. Pleural effusion analysis showed B cell lymphoma with cytogenetics showing deletion 17p13 in 10% of cells. She initially received six cycles of rituximab, cyclophosphamide, doxorubicin hydrochloride, vincristine, and prednisone (R-CHOP) from July 2011 to November 2011, followed by rituximab from January 2012 to June 2013. Five more cycles of rituximab and bendamustine infusions started in June 2017, followed by maintenance until November 2018. In June 2019, the patient also received idelalisib and rituximab. The antineoplastic venetoclax was initiated in December 2019, with the anti-CD20 Obinutuzumab added by February 2020. Chemotherapy was held when she was diagnosed with COVID-19 on January 26, 2021 (**Figure 1B**). She received a B-mAB infusion 18 d.p.i. (**Figure 1B**) but continued to do poorly. She was diagnosed with COVID-related pneumonia 20 d.p.i. and treated with oral azithromycin and ampicillin-sulbactam. She was hospitalized 23 d.p.i. (**Figure 1B**) for persistent COVID-related pneumonia and treated with intravenous solumedrol for four days, followed by eight weeks of prednisone. She continued to have shortness of breath and dyspnea with any degree of exertion. She was re-hospitalized 93 d.p.i. (**Figure 1B**) for acute hypoxemia and organizing pneumonia receiving oxygen. She received intravenous immunoglobulin due to low IgG and IgA levels 97 d.p.i. The patient continued complaining of increased shortness of breath. She was diagnosed with COVID pneumonia and chronic respiratory hypoxic failure (ARDS) and hospitalized again 119 d.p.i., where a first blood sample was taken 122 d.p.i. (**Figure 1B**) and received solumedrol for nine days. She had several admissions in the intensive care unit but improved and was eventually discharged 133 d.p.i. with prescribed daily prednisone and continued oxygen until 525 d.p.i. No other anti-cancer or immunosuppressive treatments were administered prior to a second blood sample obtained 555 d.p.i. (**Figure 1B**). The subject received BNT162b2 vaccine: 141, 169, and 238 d.p.i. (**Figure 1B**). This patient did not present antibodies against SARS-CoV-2 N protein, with notable Spike and RBD antibodies decreasing over time from mAB therapy (**Figure 1C**). Both blood samples exhibited lower than average %CD19 B-cells (**Figure 1D-E**).

**Patient 108 with solid tumor receiving C/I-mAB***.*

A 52-year-old female was diagnosed with rectal adenocarcinoma, stage IIA in 2014 and underwent a low anterior resection. She was diagnosed with a local recurrence of pulmonary involvement in December 2017 and received alkylating agent oxaliplatin, antimetabolite 5-FU, and anti-angiogenesis monoclonal antibody bevacizumab. Pathology showed KRAS mutation-positive, BRAF-negative, and NRAS-negative. The course was complicated by severe mucositis and an infusion reaction. She progressed in January 2019 and started on topoisomerase I inhibitor irinotecan, for six cycles. She underwent stereotactic ablative brachytherapy for oligometastatic disease of the lungs. The patient declined further chemotherapy. She was diagnosed with COVID-19 on September 17, 2021 (**Figure 1A-B**). She was given C/I-mAB by infusion one d.p.i. (**Figure 1B**). A blood sample was obtained 72 d.p.i. (**Figure 1B**). No other therapies or immunosuppressive treatments were administered before a second blood sample was obtained 338 d.p.i. (**Figure 1B**). Clinically she did well post-infusion. The subject did not receive any COVID-19 vaccinations. The patient exhibited anti-Spike, RBD, and N antibodies in both blood samples (**Figure 1C**) and normal B-cell levels for the second visit (no cells were recovered from visit one, **Figure 1D-E**).

**Patient 113 with solid tumor receiving C/I-mAB***.*

A 38-year-old female was diagnosed with metastatic breast cancer in June 2021. Pathology showed intraductal carcinoma with ER+, PR+, and HER 2-. She was started on paclitaxel for 12 weeks, followed by dose-dense anthracycline doxorubicin and alkylating agent, cyclophosphamide, on July 7, 2021. The subject was diagnosed with COVID-19 on August 10, 2021 during Delta infection peak (**Figure 1A-B**), and she received C/I-mAB infusion one d.p.i. (**Figure 1B**). One blood sample was obtained at 120 d.p.i. (**Figure 1B**). Subject 113 was vaccinated with the BNT162b2 vaccine: 98, 68, and 15 days pre-infection, and 106 d.p.i. (**Figure 1B**). We observed anti-Spike and RBD antibodies but no anti-N antibodies by ELISA (**Figure 1C**). Her blood sample exhibited a low % CD19 B-cells (**Figure 1D-E**), possibly a complication of paclitaxel and other cancer drug interactions^1^.

**Patient 114 with myeloma receiving C/I-mAB***.*

A 61-year-old male was diagnosed with IgG kappa multiple myeloma in May 2018. Cytogenetics showed a gain of chromosomes 7, 9, and 15. He was treated with an immunomodulatory and anti-angiogenic agent, lenalidomide, proteasome inhibitor, bortezomib, and dexamethasone starting May 2018 for four cycles, followed by maintenance lenalidomide^2^. Lenalidomide was discontinued in August 2019 because of myelosuppression. The subject was diagnosed with COVID-19 on August 24, 2021, and treated as an outpatient with C/I-mAB two d.p.i. (**Figure 1A-B**). No other cancer therapies or immunosuppressive treatments were administered, but blood samples were obtained 106, 134, and 423 d.p.i. (**Figure 1B**). The subject was vaccinated with the BNT162b2 vaccine: 14 days pre-infection and 98 d.p.i. (**Figure 1B**). We observed that anti-Spike and RBD antibodies were higher in the first two visits than anti-N IgG, but a higher anti-N on the last blood sample taken on day 421 post-mAB (**Figure 1C**). B-cells were at normal levels for all visits (**Figure 1D-E**).

**Detailed Methods**

**Study design and patient recruitment**

Patients were identified at University Medical Center New Orleans Cancer Center or inpatient oncology floor by specific prescreening criteria consisting of a cancer diagnosis in combination with a documented current infection with SARS-CoV-2 or a history of SARS-CoV-2 infection. Five patients who had received mAB therapy for SARS-CoV-2 infection were selected. At each visit, 18 mL of blood was drawn by standard venipuncture in 3 – 6 mL Na-Heparin blood tubes (BD Vacutainer, Fischer HealthCare) and processed within 24 hours. A retrospective chart review was done to collect subjects’ demographics, cancer diagnosis, cancer stage, chemotherapeutic agents, COVID-19 status, treatments for COVID-19, and vaccination status. B-mAB 35mg/mL cat# 0002-7910-01 (©Lilly USA) was obtained for antibody testing. All cases had PCR-confirmed SARS-CoV-2 infections. Control samples were used from patients with cancer (one lymphoma, four solid tumors) who had not received mAB therapy for SARS-CoV-2 infection. All control subjects had evidence of SARS-CoV-2 infection by PCR test (3/5) or anti-N-Protein specific IgG.

**Blood processing**

Blood was processed for PBMCs and plasma by density gradient centrifugation in Leukosep tubes (Greiner Bio-One) containing Ficoll-Paque PREMIUM 1.078g/ml (Cytiva)^3,4^. PBMCs were frozen at 1e7/ml in FBS with 10% DMSO. Plasma was stored at -80°C or heat-inactivated at 56°C for 30mn before testing.

**Plasma viral RNA detection**

Viral RNA load was measured by SARS-CoV-2-specific CRISPR assay targeting the nucleocapsid gene as previously reported ^5^. A positive result was considered twice the limit of viral detection, 2x10^6^ of RNA abundance expressed as the relative photoluminescence intensity of the sample.

**Antibody detection**

ELISA assay for IgG antibody to spike protein was performed using recombinant spike protein as previously described ^6^. Net ODs were calculated by subtracting background OD readings from sample values. Isotypes and subclasses of antibodies were determined using a multiplexed assay. Briefly, SARS-CoV-2 antigens (spike, RBD, N) and spike protein from seasonal CoVs (229E, OC43, HKU1, NL63; Frederick National Laboratory) were coupled to MagPlex Microspheres (Luminex) by Sulfo-NHS and EDC (Pierce) in 50 mM MES at pH 5.0. Sample plasma was diluted using PBS (1:50 for IgG1 and 1:10 for IgG2, IgG3, IgG4, IgA1, IgA2, IgM) and added to the antigen coupled MagPlex beads in 96-well plates and incubated for 2h at RT. Plates were washed 3x with 0.1% BSA and 0.05% Tween-20 in PBS. PE-conjugated subclass-specific secondary antibodies were added at 0.65µg/ml (SouthernBiotech; IgG1 [HP6001], IgG2 [HP6002], IgG3 [HP6050], IgG4 [HP6025], IgA1 [B3506B4], IgA2 [A9604D2], IgM [UHB]) for 1h at RT prior to analysis on a Luminex MagPix instrument.

**Pseudovirus neutralization assay**

Neutralization was measured using a high-throughput pseudovirus assay, as previously described ^7^. We produced similar pseudoviruses expressing functional spike proteins of SARS CoV-2 variants which included Delta, Omicron BA.1, BA.2, and BA.5. Neutralization titers were defined as the serum dilution (IC_50_) at which relative luminescence units (RLU) were reduced by 50% compared to virus control wells after subtraction of background RLUs, as determined by GraphPad Prism software.

**Fc-mediated effector function assays**

ADNKA, ADCD, and ADCP were performed as previously described^8^ except using MaxiSorp plates coated with 3 mg/mL (ADNKA) or coupled to 1mm FluoSpheres NeutrAvidin beads (Invitrogen, ADCD, ADCP) with recombinant Spike protein. All effector assays were measured using a Cytek Aurora Spectral Flow Cytometer. ADNKA was measured by gating on CD56+ CD16+ cells and analyzing %CD107a, IFNγ, and TNFα positivity. ADCD was measured by the Median Fluorescence Intensity (MFI) of C3, and the heat-inactivated MFI was subtracted from the active MFI to account for non-specific deposition. ADCP was measured by phagocytic score, which is calculated by [(percent bead-positive cells) * (MFI bead fluorescence of bead-positive cells)/100,000].

**Viral-specific T-cell analyses**

Activation Induced Marker (AIM) analyses were performed on 24h-restimulated cells as previously described ^9^ using 24h culture 0.2µg/well peptide pools. We used two sets of peptide pools (See Table S2**)**. Dynabeads Human T-Activator CD3/CD28 for T Cell Expansion and Activation (Gibco) was used as control. Post-culture, cells were harvested and stained for viability and surface markers prior to fixation (see AIM panel in **Table S2)**. Cells were washed with flow buffer (PBS 1% BSA, 0.1% Sodium Azide) and fixed for 20m at 4ºC with 10% formalin (PolyScience) before sampling on a Cytek® Aurora System. Intracellular cytokine staining (ICS) was performed to detect INFγ, IL-2, IL-17A, and IL-21 production in 5h restimulated samples with peptides. eBiosciences cell stimulation cocktail (PMA-Ion) was used as stimulation control. Brefeldin A (Life Technologies) was added after the first hour. Post-culture, cells were harvested and then stained for viability), followed by surface and intracellular staining using eBioscience intracellular fixation and permeabilization kit (ThermoFisher, ICS panel Table S2). Samples were acquired on a BD LSRFortessa™. Peptide-specific AIM or ICS analyses were normalized by subtracting untreated cells (media only).

**Data analysis**

Comparisons between the first-time point sample of each sample for mAB-treated patients vs the control group were performed using unpaired non-parametric t-tests. To evaluate the correlations between patients’ data, system serology and T-cell data, we performed a network analysis based on Spearman *rho* correlation matrices followed by Benjamin-Hochberg (BH) *P values* correction.

**Supplementary Figures**

**
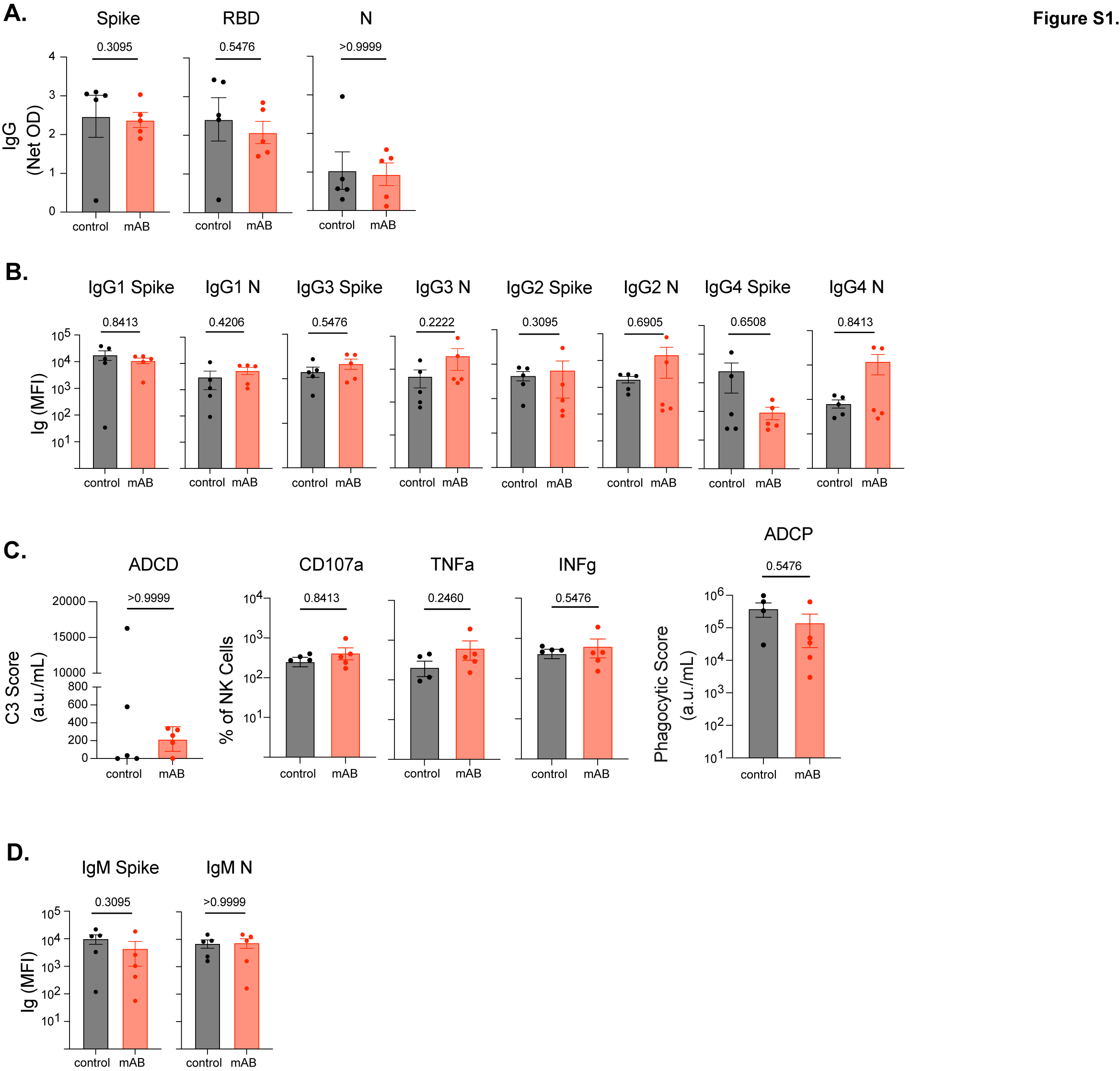
Figure S1.** **System serology comparisons between mAB-treated patients and the control group.** (A) Comparison of IgG antibodies for Spike, RBD and N by control and mAB-treated patients at first blood sample. (B) IgG isotypes comparisons for anti-Spike and anti-N. (C) Antibody effector function comparisons to spike. (D) IgM comparisons for anti-Spike and anti-N. All comparisons were performed between the first-time point sample of each sample for mAB-treated patients vs. the control group with unpaired non-parametric t-tests with *P values* indicated.

**
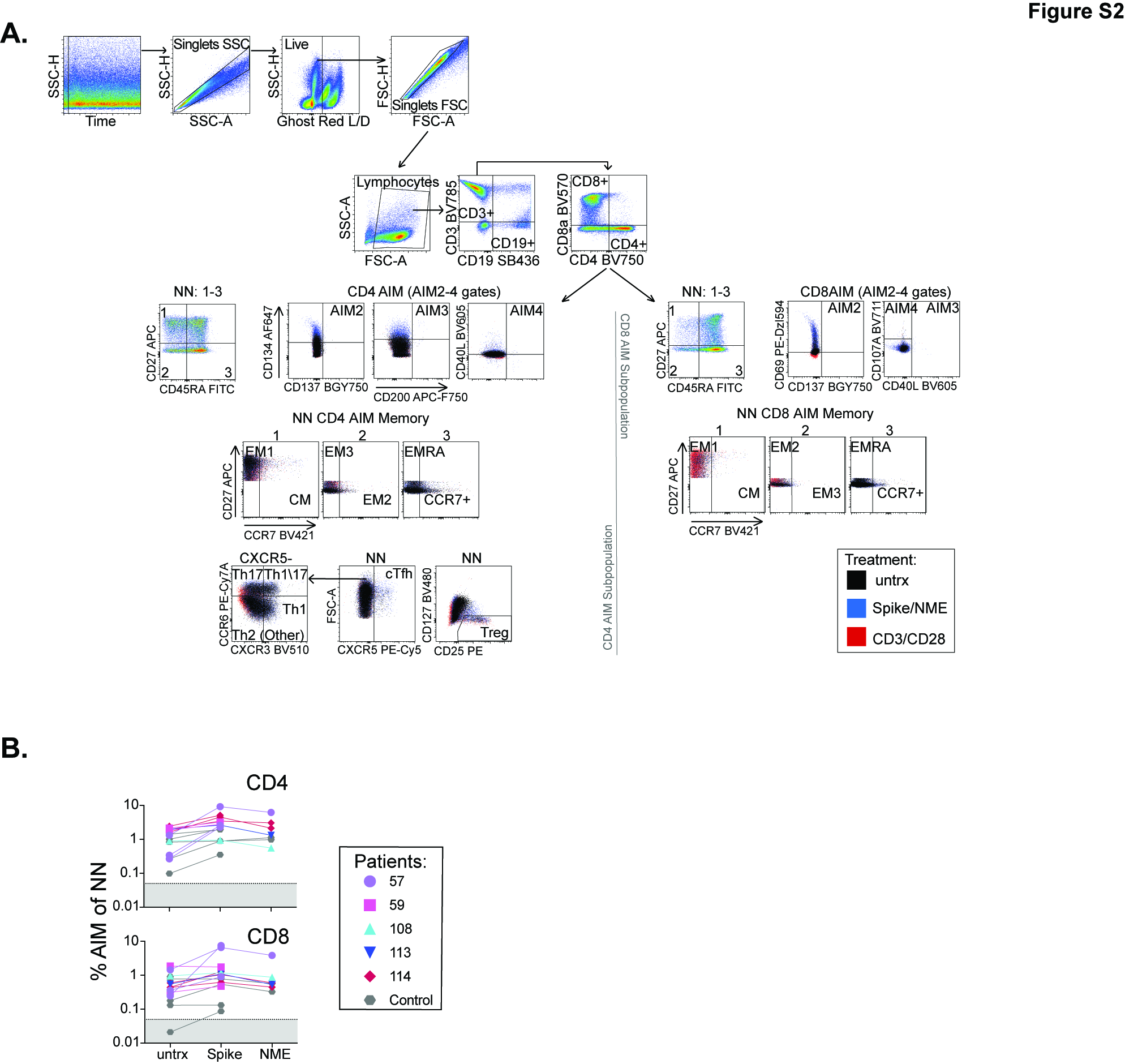
**

**Figure S2.** **Complete AIM gating strategy (20 color panel) for live cells, CD19 B-cells, and CD3 T-cells, and total % of AIM+ T-cells.** (A) Activation-induced marker (AIM) gating strategy stratified for main T-cell subsets with central memory and effector memory subsets when untreated (black) and stimulated with spike or NME (blue) peptide pools from SARS-CoV-2 and positive control CD3/CD28 beads (red). (B) The % total AIM+ CD4 or CD8 T-cells from the NN population after indicated peptide or untrx stimulation. Lines connect color-coded subject samples between individual stimulation conditions.


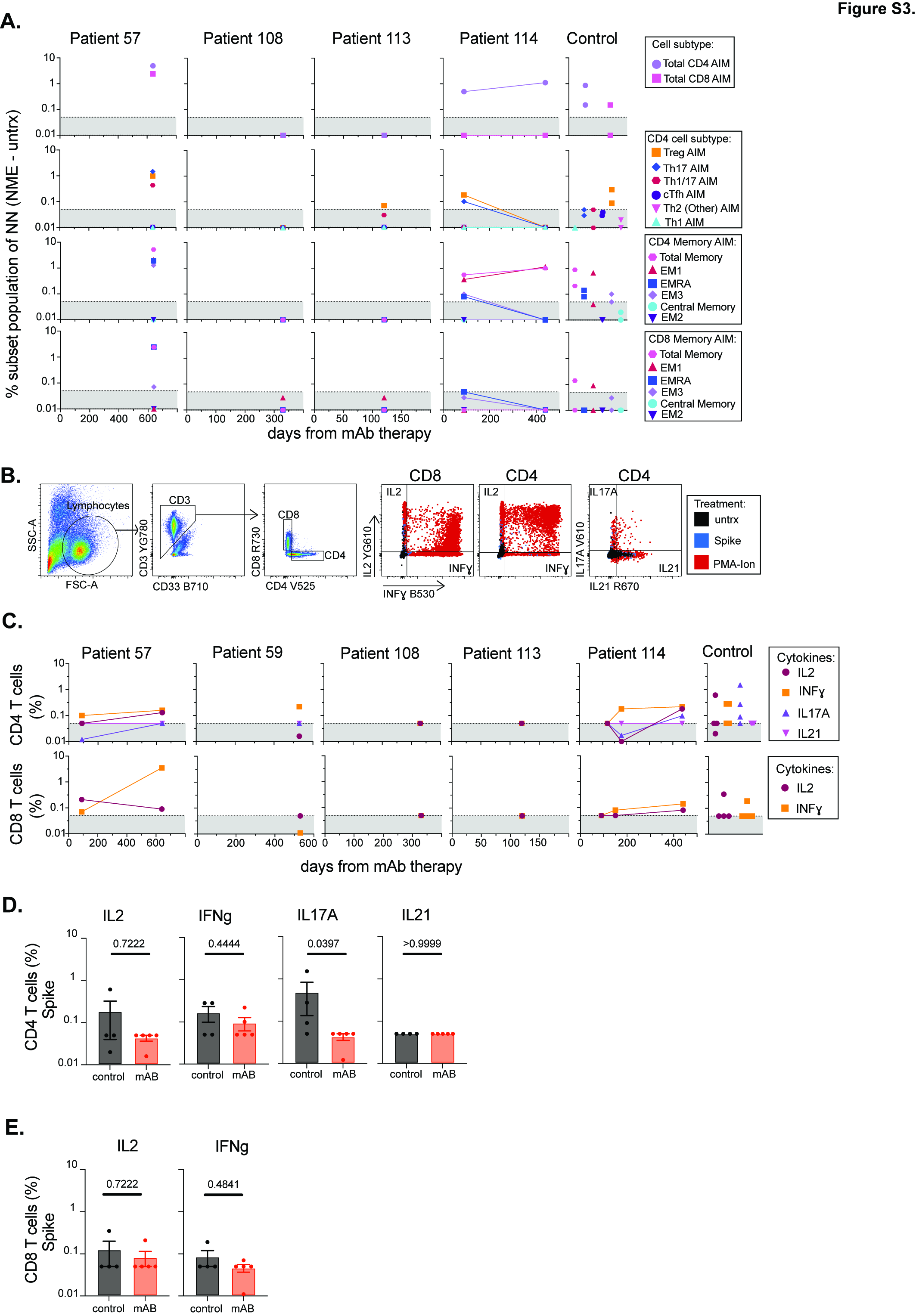


**Figure S3. Additional evidence for SARS-CoV-2-specific T-cells using non-spike AIM+ T-cells or ICS T-cell analyses.** We performed other confirmatory T-cell analyses in patients with enough PBMCs or samples. (A) Normalized non-spike (NME) specific AIM+ T-cell analysis reported as % of NN for CD4 T-cells or CD8-T cells, AIM+ CD4 subsets (Th1, Treg, etc.), and AIM+ memory states (total, EM1, etc.). (B) Spike and non-spike (NME) ICS assay gating strategy for CD4 and CD8 T-cells and indicated cytokine with untreated (untrx), spike, or mitogen (PMA-Ion) stimulation. (C) Percentage of CD4 and CD8 T-cells when stimulated with peptide pool of Spike from SARS-CoV-2 for cytokines IL-2 (brown dot), INFγ (orange square), IL-17A (violet triangle), IL-21 (pink inverted triangle). Patterned areas show the limit of detection for each assay. (D) Comparison between mAB-treated patients and control by ICS for CD4+ T-cells. (E) Comparison between mAB-treated patients and control by ICS for CD8+ T-cells. All comparisons were performed between the first-time point sample of each sample for mAB-treated patients vs. the control group with unpaired non-parametric *t-tests* with *P values* indicated.

**
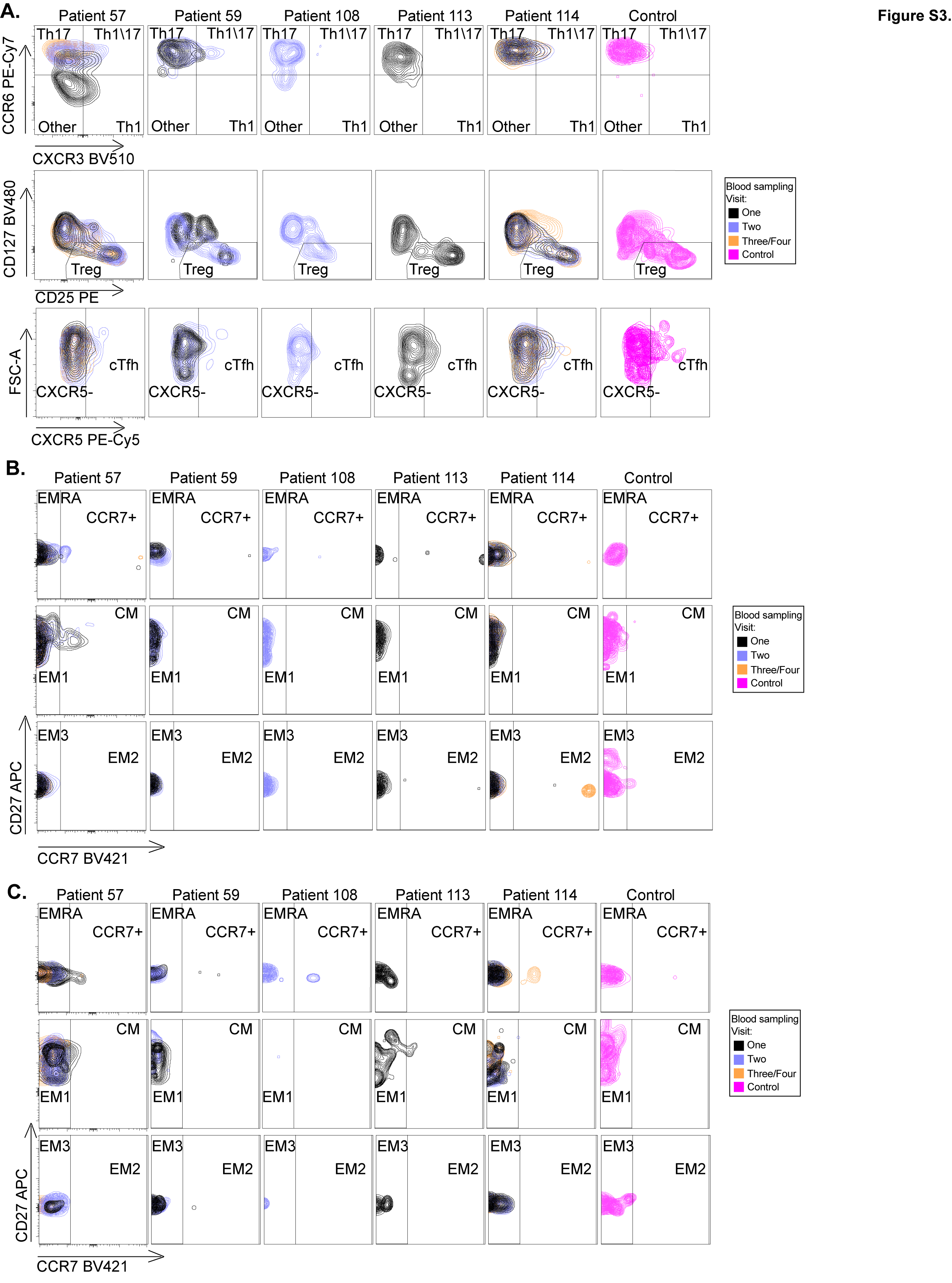
**

**Figure S4. Contour plots showing spike-stimulated CD4 and CD8 T-cells (from NN population) for indicated T cell markers for each patient by blood sample visit.** (A) Contour plots showing spike-stimulated CD4 T-cells for each CD4 T-cell subtype from the NN population. (B) Contour plots show spike-stimulated CD4 T-cells (from the NN population and not shown CD27, CD45RA analyses) memory markers for each patient by blood sampling visit. (C) Contour plots show spike-stimulated CD8 T-cells (from the NN population and not shown CD27, CD45RA analyses) memory markers for each patient by blood sampling visit.

**Table S1.** Antibody effector functions for Bamlanivimab. ADCD, ADCP, and ADNKA and viral neutralization (strain specific)*.

|  | **Bamlanivimab** | | **Isotype Control** | |
| --- | --- | --- | --- | --- |
| **Assay** | **Avg** | **STD** | **Avg** | **STD** |
| ADCD (a.u.) | 1769.42 | 10.6 | LOD | |
| ADCP (a.u.) | 4797.12 | 1474.2 | LOD | |
| ADNKA: CD107a (a.u.) | 222.56 | 20.48 | 1.92 | 2.72 |
| ADNKA: IFNγ (a.u.) | 74.06 | 15.59 | 39.76 | 5.85 |
| ADNKA: TNFα (a.u.) | 49.00 | 4.88 | 10.38 | 6.16 |
| Neutralization (Wuhan rIC50) | 143 | N/A | <50 | N/A |
| Neutralization (Delta rIC50) | <50 | N/A | <50 | N/A |
| Neutralization (BA.1, BA.2, or BA.5 rIC50) | <50 | N/A | <50 | N/A |

*Abbreviations: arbitrary units of fluorescent intensity (a.u.); limit of detection (LOC); reciprocal half-maximum inhibitory concentration for virus neutralization (rIC50); not assessed (N/A).

**Table S2.** Commercially purchased antibodies stains, and peptides for AIM and ICS flow panels.

| **PANEL** | **Fluorophore/Peptide** | **Marker** | **Cat#** | **Manufacturer** |
| --- | --- | --- | --- | --- |
| AIM | BV421 | CCR7 | 353208 | [BioLegend](https://www.biolegend.com/en-us/products/brilliant-violet-421-anti-human-cd197-ccr7-antibody-7497) |
| AIM | Super Bright 436 | CD19 | 62-0199-42 | [ThermoFisher](https://www.thermofisher.com/antibody/product/CD19-Antibody-clone-HIB19-Monoclonal/62-0199-42) |
| AIM | BV480 | CD127 | 566101 | [BD Biosciences](https://www.bdbiosciences.com/en-eu/products/reagents/flow-cytometry-reagents/research-reagents/single-color-antibodies-ruo/bv480-mouse-anti-human-cd127.566158) |
| AIM | BV510 | CD183 (CXCR3) | 353726 | [BioLegend](https://www.biolegend.com/en-us/products/brilliant-violet-510-anti-human-cd183-cxcr3-antibody-8527?GroupID=BLG6097) |
| AIM | BV570 | CD8a | 301038 | [BioLegend](https://www.biolegend.com/fr-ch/products/brilliant-violet-570-anti-human-cd8a-antibody-7371?GroupID=BLG5903) |
| AIM | BV605 | CD40L | 310826 | [BioLegend](https://www.biolegend.com/en-us/products/brilliant-violet-605-anti-human-cd154-antibody-7665?GroupID=BLG4449) |
| AIM | BV650 | PD-1 | 367430 | [BioLegend](https://www.biolegend.com/en-us/search-results/brilliant-violet-650-anti-human-cd279-pd-1-antibody-15859?GroupID=BLG5499) |
| AIM | BV711 | CD107A | 328640 | [BioLegend](https://www.biolegend.com/en-us/products/brilliant-violet-711-anti-human-cd107a-lamp-1-antibody-12003?GroupID=BLG10252) |
| AIM | BV750 | CD4 | 344644 | [BioLegend](https://www.biolegend.com/en-us/search-results/brilliant-violet-750-anti-human-cd4-antibody-15786?GroupID=GROUP28) |
| AIM | BV785 | CD3 | 317330 | [BioLegend](https://www.biolegend.com/en-us/search-results/brilliant-violet-785-anti-human-cd3-antibody-7977?GroupID=BLG4203) |
| AIM | FITC | CD45RA | 35-0458-T100 | [Tonbo](https://cytekbio.com/products/fitc-anti-human-cd45ra-hi100?variant=40581226102820) |
| AIM | PE-Dazzle 594 | CD69 | 310942 | [BioLegend](https://www.biolegend.com/en-us/products/pe-dazzle-594-anti-human-cd69-antibody-10354?GroupID=BLG10036) |
| AIM | PE | CD25 | 356104 | [BioLegend](https://www.biolegend.com/en-us/search-results/pe-anti-human-cd25-antibody-8389) |
| AIM | PE-Cy5 | CD185 (CXCR5) | 356952 | [BioLegend](https://www.biolegend.com/fr-fr/search-results/pe-cyanine5-anti-human-cd185-cxcr5-antibody-21259?GroupID=BLG10053) |
| AIM | BYG750 | CD137 | RC-00196 | [Tonbo](https://cytekbio.com/products/fitc-anti-human-cd45ra-hi100?variant=40581226102820) |
| AIM | PE-Cy7 | CCR6 | 353418 | [BioLegend](https://www.biolegend.com/en-us/search-results/pe-cyanine7-anti-human-cd196-ccr6-antibody-7564?GroupID=BLG10129) |
| AIM | APC | CD27 | 356410 | [BioLegend](https://www.biolegend.com/en-us/products/apc-anti-human-cd27-antibody-8467?GroupID=BLG7922) |
| AIM | AF647 | CD134 | 350018 | [BioLegend](https://www.biolegend.com/en-us/search-results/alexa-fluor-647-anti-human-cd134-ox40-antibody-12976) |
| AIM | (APC-Fire 750) | CD200 | 329225 | [BioLegend](https://www.biolegend.com/en-us/search-results/apc-fire-750-anti-human-cd200-ox2-antibody-15320) |
| AIM | Ghost Red 710 | Live/Dead | 13-0871-T500 | [Tonbo](https://cytekbio.com/products/fitc-anti-human-cd45ra-hi100?variant=40581226102820) |
| AIM/ICS | Human Trustain FcX |  | 422303 (200 tests) | [BioLegend](https://www.biolegend.com/en-us/products/human-trustain-fcx-fc-receptor-blocking-solution-6462) |
| ICS (surface) | BV 510 | CD4 | 317444 | BioLegend |
| ICS (surface) | AF700 | CD8 | 300920 | BioLegend |
| ICS (surface) | PE-Cy7 | CD3 | 300316 | BioLegend |
| ICS (surface) | PerCP-Cy5.5 | CD33 | 303414 | BioLegend |
| ICS | AF488 | IFNγ | 502515 | BioLegend |
| ICS | PE Dazzle 594 | IL-2 | 500344 | BioLegend |
| ICS | BV605 | IL-17A | 512326 | BioLegend |
| ICS | AF647 | IL-21 | 513006 | BioLegend |
| AIM/ICS | PepTivator® SARS-CoV-2 Prot_S |  | 130-127-951 | Miltenyi |
| AIM/ICS | PepTivator® SARS-CoV-2 Prot_N |  | 130-126-698 | Miltenyi |
| AIM/ICS | Peptide Array, SARS-Related Coronavirus 2 Membrane (M) Protein |  | NR-52403 | BEI |
| AIM/ICS | Peptide Array, SARS-Related Coronavirus 2 Envelope (E) Protein |  | NR-52405 | BEI |

**References**

1. Javeed A, Ashraf M, Riaz A, Ghafoor A, Afzal S, Mukhtar MM. Paclitaxel and immune system. *Eur J Pharm Sci*. Nov 5 2009;38(4):283-90. doi:10.1016/j.ejps.2009.08.009

2. Pellom ST, Jr., Dudimah DF, Thounaojam MC, Sayers TJ, Shanker A. Modulatory effects of bortezomib on host immune cell functions. *Immunotherapy*. 2015;7(9):1011-22. doi:10.2217/imt.15.66

3. Mallone R, Mannering SI, Brooks-Worrell BM, et al. Isolation and preservation of peripheral blood mononuclear cells for analysis of islet antigen-reactive T cell responses: position statement of the T-Cell Workshop Committee of the Immunology of Diabetes Society. *Clinical and Experimental Immunology*. 2010;163(1):33-49. doi:10.1111/j.1365-2249.2010.04272.x

4. Nilsson C, Aboud S, Karlén K, Hejdeman B, Urassa W, Biberfeld G. Optimal blood mononuclear cell isolation procedures for gamma interferon enzyme-linked immunospot testing of healthy Swedish and Tanzanian subjects. *Clin Vaccine Immunol*. Apr 2008;15(4):585-9. doi:10.1128/cvi.00161-07

5. Ning B, Huang Z, Youngquist BM, et al. Liposome-mediated detection of SARS-CoV-2 RNA-positive extracellular vesicles in plasma. *Nat Nanotechnol*. Sep 2021;16(9):1039-1044. doi:10.1038/s41565-021-00939-8

6. Drouin AC, Theberge MW, Liu SY, et al. Successful Clearance of 300 Day SARS-CoV-2 Infection in a Subject with B-Cell Depletion Associated Prolonged (B-DEAP) COVID by REGEN-COV Anti-Spike Monoclonal Antibody Cocktail. *Viruses*. Jun 23 2021;13(7)doi:10.3390/v13071202

7. Iwanaga N, Cooper L, Rong L, et al. ACE2-IgG1 fusions with improved in vitro and in vivo activity against SARS-CoV-2. *iScience*. Jan 21 2022;25(1):103670. doi:10.1016/j.isci.2021.103670

8. Gunn BM, Lu R, Slein MD, et al. A Fc engineering approach to define functional humoral correlates of immunity against Ebola virus. *Immunity*. Apr 13 2021;54(4):815-828 e5. doi:10.1016/j.immuni.2021.03.009

9. Painter MM, Mathew D, Goel RR, et al. Rapid induction of antigen-specific CD4(+) T cells is associated with coordinated humoral and cellular immunity to SARS-CoV-2 mRNA vaccination. *Immunity*. Sep 14 2021;54(9):2133-2142 e3. doi:10.1016/j.immuni.2021.08.001
